# Senescence explains age- and obesity-related liver steatosis

**DOI:** 10.15698/cst2017.10.108

**Published:** 2017-09-19

**Authors:** Mikolaj Ogrodnik, Diana Jurk

**Affiliations:** 1 Institute for Ageing, Institute for Cell and Molecular Biosciences (ICaMB), Campus for Ageing and Vitality, Newcastle University, Newcastle upon Tyne, NE4 5PL, UK.

**Keywords:** NAFLD, senescence, senolytic, ageing

## Abstract

Cellular senescence, the irreversible loss of replicative potential of somatic cells, was first described in fibroblasts cultured *in vitro *by Leonard Hayflick more than 50 years ago. Since then, the field of cellular senescence has witnessed a meteoric rise, with multiple studies highlighting its importance in varied physiological contexts such as cancer, development and ageing. A major recent development in the senescence field has been the creation of mouse models which allow the specific elimination of senescent cells. These genetic tools have allowed scientists, for the first time, to conduct proof-of-principle investigations into the causal impact of senescence during the ageing process and in the context of several age-related diseases. Furthermore, these experiments provided the rationale for the development of a new class of drugs named “senolytics”, that can specifically kill senescent cells, which are now of great interest to academics and pharma companies alike. Non-alcoholic fatty liver disease (NAFLD) is more prevalent in the older and obese population and unrelated to alcohol consumption. It can be characterized by simple liver fat accumulation (steatosis) but it can progress to more severe stages such as non-alcoholic steatohepatitis (NASH), advanced fibrosis, cirrhosis and hepatocellular carcinoma (HCC). Previous studies have demonstrated that during ageing and NAFLD, hepatocytes accumulate markers of cellular senescence. However, until now, it was unclear whether senescence was a cause or consequence of liver disease.

In a study recently published in the journal *Nature Communications* we have researched the role of cellular senescence during liver ageing, in obesity and NAFLD. We observed that hepatocyte fat accumulation occurred in parallel with markers of cellular senescence in mice exposed to different dietary regimes and in human patients with different severities of non-alcoholic fatty liver disease. This initial observation led us to postulate that hepatic steatosis may be a consequence of hepatocyte senescence.

To investigate this hypothesis, we first utilised the Ink-ATTAC mouse model, in which a small molecule AP20187 can induce apoptosis in p16^Ink4a^-postive cells, thereby eliminating them from tissues. We found that drug-induced elimination of p16^Ink4a^-senescent cells in both aged and high-fat fed Ink-ATTAC mice resulted in a significant reduction in liver fat accumulation. Secondly, we investigated the potential role of senolytic drugs as a therapy against liver steatosis. We found that treatment with a senolytic cocktail of dasatinib and quercetin (D+Q) significantly reduced liver fat accumulation in aged wild-type mice and in obese mice (*db/db*), which carry a mutation in the leptin receptor (Figure 1).

**Figure 1:**
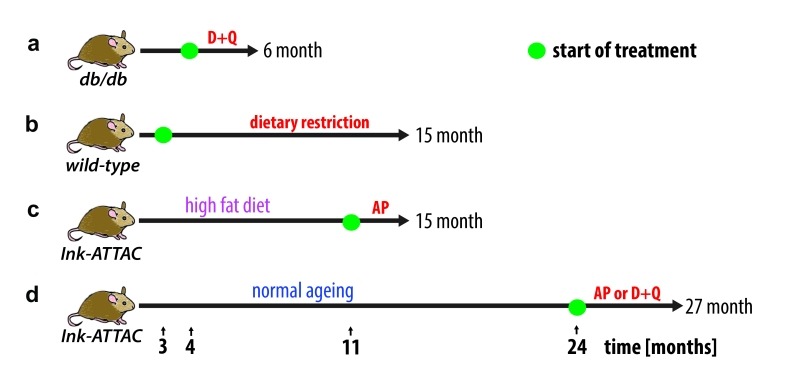
FIGURE 1: Treatment scheme for obese and ageing animals. **(A)*** db/db* mice were treated with D+Q for 2 months until 6 months of age. **(B)** Wild-type mice underwent several dietary restriction regimes. **(C)** Ink-ATTAC mice under high fat diet were treated with AP20187 for 4 months and **(D)** ageing Ink-ATTAC mice were treated for 3 months wit AP20187 or D+Q.

It remains a possibility that the observed reduction in liver fat is the result of non-cell autonomous effects, since none of the previously described interventions is specifically targeting senescent hepatocytes. In fact, previous studies using both Ink-ATTAC mice and senolytic drugs have shown that drug-induced elimination of senescent cells occurs simultaneously in multiple tissues and ameliorates multiple ageing phenotypes. Thus, it is reasonable to assume that reduced liver fat accumulation may be partially the outcome of clearance of senescent cell-types other than hepatocytes.

In order to investigate this further, we used a mouse model in which senescence can be induced specifically in hepatocytes via inactivation of the DNA repair gene Xpg. We found that hepatocytes lacking Xpg exhibited increased senescent markers as well as increased fat accumulation. These data support the hypothesis that cell senescence results in fat accumulation in a cell autonomous fashion.

Mechanistically it is still unclear why senescent hepatocytes accumulate fat. Previous data suggests that factors such as high fat diet, increased lipid synthesis or decreased lipid catabolism may contribute to steatosis during NAFLD. Hepatic fat accumulation may promote the progression of NAFLD to more severe stages by promoting inflammation, fibrosis and cell death.

Data from our group and others have shown that senescent cells have dysfunctional mitochondria, characterized by impaired oxidative phosphorylation and increased generation of Reactive Oxygen Species (ROS). In fact, recent data indicates that mitochondria are an integral part of the senescence program, since ablation of mitochondria alleviates many of the senescent phenotypes.

Since mitochondria are the major sites of fatty acid oxidation, we proposed that impaired fatty acid oxidation during senescence could explain fat accumulation. Consistent with this hypothesis, we found that mitochondria from senescent fibroblasts and hepatocytes were less efficient in metabolizing the fatty acid palmitate and were insensitive to inhibition of fatty acid oxidation by etomoxir when compared to young cells (Figure 2).

**Figure 2:**
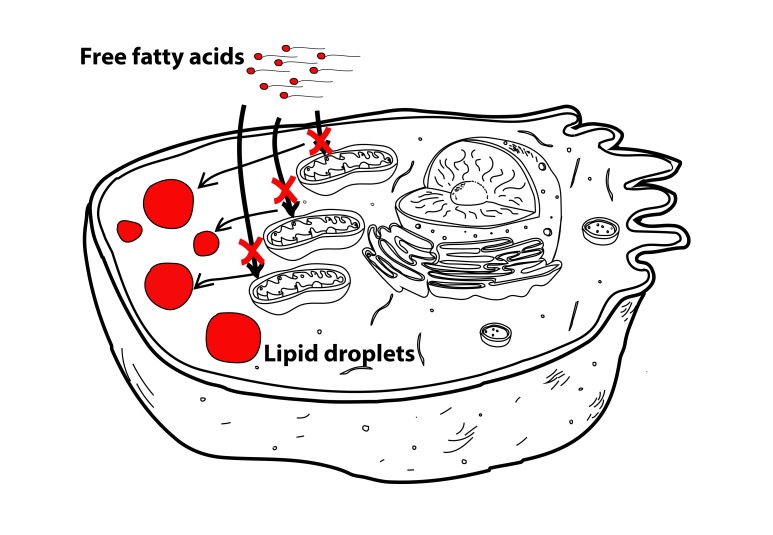
FIGURE 2: Impaired fatty acid oxidation in senescent cells leads to increased fat storage. Induction of cellular senescence induces mitochondrial dysfunction and impaired fatty acid oxidation, thus contributing to increased cytosolic fat accumulation (lipid droplets).

While our study indicates that excessive fat accumulation is a consequence of hepatocyte senescence, it does not answer why hepatocyte senescence is induced in the first place. Telomere damage, increased fatty acids, mitochondrial dysfunction and chronic inflammation are associated with ageing, obesity and liver disease and have been shown to induce cellular senescence *in vitro* and *in vivo*. Furthermore, all these factors have been shown to be modulated by dietary interventions known to impact on hepatic steatosis. Given the complexity and interconnected nature of these processes we speculate that it is unlikely that one single mechanism is driving hepatocyte senescence and that probably a combination of multiple factors is at play.

In conclusion, ageing and obesity are major risk factors for a variety of age-related disease including NAFLD. Our study demonstrates that cellular senescence is a major driver of hepatic steatosis. Furthermore, it highlights that elimination of senescent cells may be a novel therapeutic strategy to reduce steatosis, thereby ameliorating the progression of NAFLD.

In summary, we have revealed a strong association between cellular senescence and liver fat accumulation. Dietary restriction (DR) decreased numbers of senescent cells in parallel to strong inhibition of liver fat accumulation or even fat reduction. Using genetic and drug interventions we were further able to replicate some beneficial effects of DR on liver fat storage. The novelty of our approach is however not in inhibiting accumulation of senescent cells, but in alleviating liver steatosis through direct elimination of senescent cells that have already accumulated. As we took only the first step in understanding the mechanistic basics, it is to future research to elucidate other aspects of this phenomenon.

